# Prevalence and molecular characterization of *Strongyloides* spp. in domestic and wild felids from Switzerland

**DOI:** 10.1016/j.ijppaw.2026.101284

**Published:** 2026-09-05

**Authors:** Gastón Moré, Giulia Colangeli, Simone R.R. Pisano, Fatou Geissler, Iris Marti, Sinah Lückner, Chahrazed Belhout, Diana S. Gliga, Caroline F. Frey, Walter Basso

**Affiliations:** a Institute of Parasitology, Vetsuisse-Faculty, University of Bern, Länggassstrasse 122, Bern, 3012, Switzerland; b Institute for Fish and Wildlife Health, Vetsuisse-Faculty, University of Bern, Länggassstrasse 122, Bern, 3012, Switzerland; c Institute of Veterinary Bacteriology, Vetsuisse-Faculty, University of Bern, Länggassstrasse 122, Bern, 3012, Switzerland; d Institute for Infectious Diseases (IFIK), University of Bern, Bern, Switzerland; e Multidisciplinary Center for Infectious Diseases (MCID) of the University of Bern, Switzerland

**Keywords:** *Strongyloides planiceps*, *cox1*, *18S* rRNA, Oxford nanopore sequencing, Deep amplicon sequencing, Eurasian lynx

## Abstract

*Strongyloides* spp. infecting felids remain largely understudied. To date, four *Strongyloides* species have been reported in felids worldwide: *S. felis*, *S. planiceps*, *S. stercoralis*, and *S. tumefaciens*. Most epidemiological studies performed in felids identified the parasites only to genus level. This study aimed to determine the prevalence of *Strongyloides* spp. in domestic cats (*Felis catus*), free-ranging Eurasian lynx (*Lynx lynx*), and European wildcats (*Felis silvestris*) from Switzerland, while providing detailed morphological and molecular characterization of the parasites. A total of 490 intestinal content and faecal samples (172 domestic cats, 261 lynx, and 57 wildcats) were analysed using Baermann and sedimentation-flotation techniques. Morphological measurements of *Strongyloides* spp. eggs, larvae and adults were recorded. Molecular identification involved conventional PCRs targeting the *18S* rRNA and *cox1* gene fragments, followed by Sanger sequencing, Oxford Nanopore Technologies (ONT) deep amplicon sequencing and bioinformatic analysis. The coproscopical prevalence of *Strongyloides* spp. in wild felids was 9.6% (25/261) in lynx and 7% (4/57) in wildcats, whereas in domestic cats it was 0.6% (1/172). All positive samples contained free first-stage rhabditiform larvae. Eight lynx samples additionally had larvated eggs and in three lynx and one wildcat, also rhabditiform adults were present. Morphological features were consistent with *S. planiceps*. Sanger sequencing mostly failed. ONT sequencing and phylogeny confirmed *S. planiceps* in 16 representative samples (12 lynx, three wildcats, one domestic cat) and revealed high intraspecific variability in the *cox1* gene. Additionally, *S. ratti* sequences were detected in one wildcat. This study represents the first prevalence study with molecular characterization in wild felid populations globally, and the first identification of *S. planiceps* in Europe. A higher prevalence was observed in wild felids than in domestic pet cats. ONT deep amplicon sequencing proved to be a robust tool for identifying *Strongyloides* species from complex samples, overcoming the Sanger sequencing limitations.

## Highlights

• Faecal samples of 490 felids were analysed by Baermann and sedimentation-flotation.
• *Strongyloides* prevalence was 9.6% in lynx, 7% in wildcats, and 0.6% in domestic cats.
• All positive samples (n = 30) contained first-stage rhabditiform larvae.
• The morphology of eggs, larvae and adults was consistent with *Strongyloides planiceps*.
• ONT deep amplicon sequencing of *18S* and *cox1* allowed confirmation of *S. planiceps*.

## Graphical abstract

**Figure:**
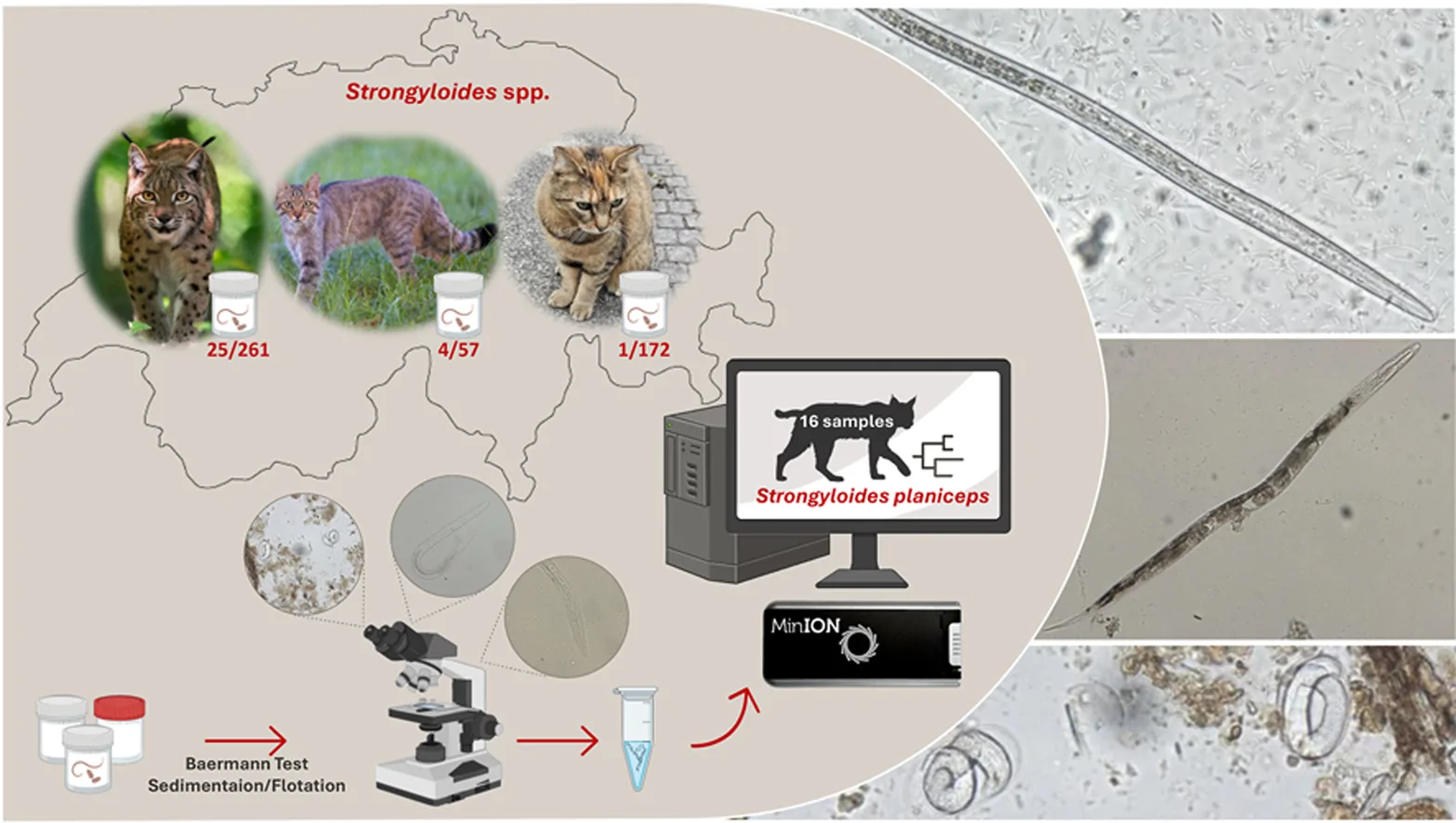

## 1 Introduction

*Strongyloides* (order Rhabditida, family Strongyloididae) is a genus of parasitic nematodes capable of infecting a wide variety of vertebrates, including humans and other primates, companion animals such as dogs (*Canis lupus familiaris*) and cats (*Felis catus*), as well as a broad range of wild mammals (Viney, 2017).

The life cycle of *Strongyloides* spp. differs from that of other nematodes, as it alternates between parasitic and free-living developmental phases. Transmission occurs mainly percutaneously, when infective filariform third-stage larvae (L3) penetrate the host's skin. After penetration, the larvae migrate either directly through the bloodstream or via the pulmonary-tracheal route to the small intestine (Tindall and Wilson, 1988). During migration, the larvae moult to the filariform fourth larval stage (L4) and subsequently develop into parasitic adult females in the small intestine. These females reproduce parthenogenetically within the intestinal mucosa of the host. Depending on the species, either larvated eggs or first-stage rhabditiform larvae (L1) are released with the faeces into the environment (Thamsborg et al., 2017). In the environment, further development can follow two alternative pathways. In the direct (homogonic) cycle, the L1 develop through the second-stage rhabditiform larvae (L2) into infective filariform L3. Alternatively, in the indirect (heterogonic) cycle, L1 develop through several stages into free-living adult males and females that mate and produce a new generation of infective L3 (Basso et al., 2019; Viney and Morris, 2022).

*Strongyloides* spp. infecting felids remain largely understudied, particularly regarding their prevalence, epidemiology, and clinical relevance. To date, four *Strongyloides* species have been reported in felids: *S. felis, S. stercoralis, S. planiceps,* and *S. tumefaciens* (Zhao and Bradbury, 2024). *Strongyloides felis* (Chandler, 1925) was the first species identified in domestic cats (*Felis catus*). Based on morphological and biological characteristics, Chandler had already suggested that this species might represent a subspecies of *Strongyloides stercoralis.* This close evolutionary relationship was later supported by others (Jitsamai, 2019; Ko et al., 2020). Although some studies have reported *S. stercoralis* infection in cats, methods used for identification at species-level were rarely provided (Zhao and Bradbury, 2024; Wulcan et al., 2019). *Strongyloides planiceps* was first identified by Leiper in 1927 in flat-headed cat *(Prionailurus planiceps)* from Malaysia and further described by Rogers (Rogers, 1939). Later, *S. planiceps* was also found in other wild carnivores like raccoon dogs (*Nyctereutes procyonoides*) and Japanese weasels (*Mustela sibirica itatsi*), as well as in domestic cats from Japan (Fukase et al., 1985; Thamsborg et al., 2017; Ko et al., 2020). *Strongyloides tumefaciens* was initially identified in two domestic cats from the southeastern United States and was characterized by its ability to induce colonic nodular lesions observed at necropsy (Price and Dikmans, 1941). However, similar lesions have also been described in cats infected with *S. stercoralis* (Wulcan et al., 2019). Genetically similar *S. stercoralis* isolates are associated with zoonotic transmission and have been reported in dogs, humans, and non-human primates, thereby raising questions about the taxonomic validity of *S. tumefaciens* (Wulcan et al., 2019).

Several morphological key characters are used to identify *Strongyloides* species (Viney, 2017). *Strongyloides* spp. eggs can be identified by their thin shell, ellipsoidal shape, slightly flattened poles, and the presence of an underdeveloped rhabditiform larva at the time of shedding. Their length typically ranges from 40 to 55 μm, with a width corresponding to approximately one-half to three-quarters of the length (Zhao et al., 2026). Rhabditiform larvae can belong to four different stages (L1-L4). They are generally differentiated by their size, which increases over time, the L1 measuring between 200 and 380 μm in length depending on the *Strongyloides* species (Zhao et al., 2026). No morphological features allow differentiation among *Strongyloides* spp. infective L3; however, they can be distinguished from other nematode larvae by their slender shape, elongated filariform oesophagus, and a notched tail tip, which is considered characteristic for the genus. Generally, they measure around 400–700 μm in length and 12–20 μm in width, with the filariform oesophagus occupying approximately 40–45% of the body length (Zhao et al., 2026). It has been highlighted that *S. planiceps* differs from other *Strongyloides* species affecting felids. In particular, the parasitic female can be distinguished from those of *S. felis* and *S. stercoralis* by two main traits: the ovaries, which are spiralled rather than straight, and the tail, which is bluntly rounded rather than narrowly tapered (Zhao et al., 2026). The free-living male also differs from these species, most notably in the spicule ventral membrane, which is prominent in *S. planiceps* but inconspicuous in *S. felis* and *S. stercoralis* (Zhao et al., 2026). Additionally, *S. planiceps* stands out from other species due to certain biological features, particularly the presence of larvated eggs, rather than larvae, in fresh faeces (Rogers, 1939), and its capacity to produce up to nine consecutive free-living generations (Yamada et al., 1991), whereas in *S. stercoralis* and *S. felis* the free-living phase is limited to a single generation (Zhao et al., 2026). However, similarities among certain species make morphological identification alone unreliable; therefore, combination with genotyping has become fundamental (Zhao et al., 2026). Over the past two decades, genotyping approaches have been developed and applied to compare strains from different isolates (Bradbury et al., 2021). The most commonly sequenced markers include the hypervariable regions I and IV of the *18S* ribosomal RNA coding gene (rDNA) and selected regions of the cytochrome *c* oxidase subunit I gene of mitochondrial DNA (*cox1*) (Hasegawa et al., 2009; Ko et al., 2020; Bradbury et al., 2021; Zhao et al., 2026). PCR followed by Sanger sequencing was mainly applied, but also next generation sequencing (NGS), specifically deep amplicon sequencing with Illumina MiSeq platform has been used (Zhao et al., 2026). This last approach is particularly useful for complex samples like faeces or when multiple parasite species or genotypes are present (Šlapeta et al., 2026; Zhao et al., 2026).

A recent systematic review identified a pooled *Strongyloides* spp. global prevalence of 12.2% in domestic cats and 20.0% in wild felids. These positivity rates are higher than the *S. stercoralis* prevalence in humans (8.1%) and dogs (6%) (Zhao and Bradbury, 2024). Feline strongyloidosis has a worldwide distribution, with highest pooled prevalence in Africa and the Western Pacific (around 47-50%). Studies across several countries have reported higher prevalences in stray cats (6.3–52.1%) than in pet (3.7–14.9%) and shelter cats (0–9.0%) (Zhao and Bradbury, 2024). Eggs or larvae attributed to the genus *Strongyloides* have been detected in cat faeces from multiple countries, including Bangladesh, Brazil, Bulgaria, Denmark, Egypt, England, France, Italy, Myanmar, and Nepal (Zhao and Bradbury, 2024). Domestic cats from Australia and Thailand have been reported to be infected with *S. felis* (Speare and Tinsley, 1987; Jitsamai, 2019). In the study performed in Australia, a relatively high prevalence was detected in domestic cats (33.5% of 504 cats), based on the detection of rhabditiform larvae in the Baermann funnel test (Speare and Tinsley, 1987). *Strongyloides stercoralis* has been identified in domestic cats from Brazil, Italy, Kenya, Qatar and the United States of America, but the methods for species confirmation were not specified (Zhao and Bradbury, 2024). Regarding *S. planiceps*, infected cats have been reported almost exclusively in Japan (Zhao et al., 2026). Experimental infections in domestic cats have been performed using isolates of *Strongyloides* spp. and *S. planiceps* derived from dogs and wild carnivores, respectively. Subsequently, larvated eggs were detected in the faeces, suggesting the presence of *S. planiceps* in both cases (Fukase et al., 1985; Zhao et al., 2026). *Strongyloides* spp. have been reported in several wild felid species in North and South America, and in Europe. Specifically, their presence has been investigated in bobcats (*Lynx rufus*) in the USA (Heidt et al., 1988); pumas (*Puma concolor*) in the USA, Peru, and Mexico (Foster et al., 2006; Aranda et al., 2013; Solórzano-García et al., 2017); jaguars (*Panthera onca*) in Peru and Mexico (Aranda et al., 2013; Solórzano-García et al., 2017); ocelots (*Leopardus pardalis*) and margays (*Leopardus wiedii*) in Peru (Aranda et al., 2013); and European wildcats (*Felis silvestris*) in Croatia (Martinković et al., 2017). All these studies performed coproscopical examinations and detected either adult parasites, rhabditiform larvae or larvated eggs; however, no identification to species level was conducted (Zhao and Bradbury, 2024). To date, however, no data have been published on the occurrence of *Strongyloides* spp. in the Eurasian lynx (*Lynx lynx*).

The Eurasian lynx has a broad distribution across Eurasia, covering a wide latitudinal range from Europe to Asia. However, in Central and Western Europe the species only occurs in a few highly fragmented populations. In Switzerland, the Eurasian lynx became extinct during the 19th century (Breitenmoser, 1998). Reintroduction efforts began in 1971, with releases of individuals helping to establish lynx populations in the Jura mountains and in the Swiss Alps (Breitenmoser and Breitenmoser-Würsten, 2008; Linnell et al., 2009). The population in the Jura mountains is separated from the Alpine populations by the highly human-dominated landscape of the Swiss Midland Plateau (thereafter Midlands). However, the presence of lynx in the Midlands has increased in recent years (Foundation KORA, 2021). According to the most recent monitoring reports, during the biological year 2024–2025 the Swiss lynx population was estimated at 364 (±10) independent lynx (subadults and adults, i.e. individuals older than one year), with over two thirds of individuals living in the Alpine populations (https://www.kora.ch/en/species/lynx/abundance, accessed 20 July 2026).

The European wildcat (*Felis silvestris*) was once widespread throughout Europe until the 19th century, when its population drastically declined. In Switzerland, over the last three decades, the species has slowly returned to its original distribution in the Jura Mountains and recently started to recolonize the Midlands (www.kora.ch, retrieved 20th July, 2026) The latest estimate of the Swiss wildcat population reported approximately 1100 individuals in 2020 and a density of 26 (17–36) wildcats per 100 km^2^ of suitable habitat was estimated in the northern Jura Mountains (Maronde et al., 2020).

Regarding domestic cats (*Felis catus*), an estimated 1.8 million individuals are present in Switzerland, with around 1.52 million being domestic pet cats and the rest represented by stray cats roaming freely (https://www.vhn.ch/de/statistiken, accessed 20 July, 2026). This relatively large domestic cat population and the distinctive proximity of cats to humans have prompted investigation on the presence of zoonotic parasites in faecal material and intestinal content, where *Strongyloides* sp. was detected in one of the analysed samples, but the species was not identified (Furtado Jost et al., 2023).

The aims of this study were to identify the prevalence of *Strongyloides* spp. in domestic cats and wild felids from Switzerland, as well as to perform their morphological and molecular characterization using Sanger and deep amplicon sequencing.

## 2 Materials and methods

### 2.1 Sample collection

A total of 490 intestinal content and/or faecal samples from domestic cats (n = 172) and wild felids (n = 318) were received between August 2017 and December 2025 at the Institute of Parasitology, *Vetsuisse* Faculty, University of Bern (IPB). The samples from live domestic pet cats (n = 81) were submitted by veterinarians to routine diagnostics, while the deceased (n = 91) animals were collected and sampled within the framework of another study (Furtado Jost et al., 2023). Geographically, cat samples originated mainly from urban environments in the central-western region of Switzerland, namely Midlands and the Jura regions.

Wild felid samples were obtained from Eurasian lynx (n = 261) and wildcats (n = 57). Most of the samples (255/261 lynx and 54/57 wildcats) derived from free-range animals and were obtained either during standardized postmortem examination performed at the Institute of Fish and Wildlife Health (FIWI), Vetsuisse Faculty, University of Bern, within the framework of the Swiss national wildlife health surveillance programme (Ryser-Degiorgis and Segner, 2015; Scherrer et al., 2023) (n = 305), or during handling of live lynx during translocation programmes (n = 4). The coordinates of the sites where the carcasses were found or live animals were captured were recorded. The carcasses were submitted to the FIWI usually within hours after detection, and kept at 4-8°C temperature until the necropsy was performed. Necropsy were usually performed within 12 h after carcass reception and the intestinal content was processed within 24 h after collection. The remaining nine samples from captive wild felids (6 Eurasian lynx, 3 wildcats) were submitted from different zoological and animal parks from Switzerland. Of all samples from free-ranging wild felids with known geographical origin (n = 302), 107 (35.4%) originated from Jura, 87 (28.8%) from the Alps, 82 (27.1%) from the Midlands, and 26 (8.6%) from the Prealps region.

Three age classes were considered for wild felids: juveniles, subadults and adults based on dentition, morphometric characteristics or birth date when available (Marti and Ryser-Degiorgis, 2018a; 2018b). Free-ranging lynx were grouped as juveniles (<1 year): n = 162; subadults (≥1 year to <2 years for females and ≥ 1 year to <3 years for males): n = 35; and adults (≥2 years for females and ≥ 3 years for males): n = 58. Wildcats were classified as juveniles n = 7; subadults n = 4; and adults n = 43. Captive animals consisted of 4 adult lynx and one juvenile lynx and two adults and one juvenile wildcat. The age was not informed for one captive lynx.

### 2.2 Microscopical examination

Faecal sedimentation–flotation and the Baermann test were performed to detect *Strongyloides* spp. eggs and larvae, respectively, as previously described (Basso et al., 2019). Baermann sediments were microscopically examined at 100× and 400× total magnification, and *Strongyloides-*like stages were documented (Nikon Eclipse Ci with a calibrated Nikon Camera model DFK 23UP031). For most positive samples, sediment recovered from the Baermann test was stored at −20°C for subsequent molecular analysis. In a few samples, and in order to obtain more purified material for DNA extraction, the recovered larvae were washed three times with phosphate-buffered saline (PBS) prior to further processing.

### 2.3 Statistical analysis

The comparison of positivity rate in the coproscopical analysis between and among felids species, age categories and regions of origin of wild felids was conducted by chi2 for differences of percentages using the online tool Working in Epidemiology (http://www.winepi.net/uk/index.htm).

### 2.4 Necropsy and histopathology

Standardized necropsy and histopathological examination were conducted in all the free-ranging lynx and wildcats received at FIWI within the framework of the national wildlife health surveillance programme. Sampled tissues were fixed in 4% buffered formalin and embedded in paraffin to finally be cut to 5 μm slices and stained with haematoxylin and eosin (HE). Emphasis was placed on examining sections of the lung and small intestine from all animals, which tested positive for *Strongyloides* spp. by coprological analyses, with the aim of identifying migrating larvae and/or adult parasitic female nematodes, respectively.

### 2.5 Molecular studies

#### 2.5.1 DNA extraction

Approximately 150 μL of Baermann sediment containing larvae or washed larvae from individual positive samples in microscopy were subjected to DNA extraction using the Quick-DNA™ Fecal/Soil Microbe Miniprep Kit (Zymo Research, USA), according to the manufacturer's instructions. Mechanical lysis was performed using a TissueLyser II (QIAGEN, USA) at 30 Hz for 10 min.

#### 2.5.2 Polymerase chain reactions (PCRs) and Sanger sequencing

Two different Hyper Variable Regions (HVRs) from the small subunit ribosomal RNA gene (*18S*) and a fragment of the mitochondrial cytochrome *c* oxidase subunit I (*cox1*) gene of *Strongyloides* spp. were amplified using different conventional PCR assays. The positive PCR products were subsequently sequenced.

*18S* PCRs: conventional PCRs were performed to amplify the Hypervariable Region I (HVR I) and the HVR IV of *Strongyloides* spp. The HVR I was amplified using the primers SSU18A (5′-AAAGATTAAGCCATGCATG-3′) and SSU26R (5′-CATTCTTGGCAAATGCTTTCG-3′) (product ∼860 bp) described by Jaleta et al. (2017), while HVR IV was amplified using the primers ENM280 (5′-CTGCGTGAGAGGTGAAAT-3′) and ENM281 (5′-GCTACCTTGTTACGACTTTT-3′) as described by Holterman et al. (2006). Both PCR assays were performed on a Flexlid Mastercycler (Eppendorf, Hamburg, Germany) under the following conditions: for HVR I 94°C for 15 min, 35 cycles (94°C for 30 s, 52°C for 15 s and 72°C for 90 s), and final extension 72°C for 10 min; and for HVR IV 94°C for 15 min, 40 cycles (94°C for 30 s, 53°C for 30 s, 72°C for 35 s); and final extension 72°C for 5 min.

*cox1* PCR: Initially the samples were screened by a conventional PCR using the primers COI int F and COI int R proceeding as previously described (Basso et al., 2019). Additionally, two other PCRs were conducted targeting a ∼950 bp fragments of the *cox1* gene using the forward primer ENM282 (5′-CTATTTTAACTGCTCATGCT-3′) and the reverse primer ENM310 (5′-CTAACTACATAATAAGTATCATG-3′) or using the same forward primer with the reverse primer ENM283 (5′-ACCATGTAAACCAGCAAAATG-3′) (Ko et al., 2020). The same thermal cycler previously mentioned was used with the following conditions for both primer combinations: 94°C for 15 min, 40 cycles (at 94°C for 45 s, 52°C for 45 s, and 72°C for 90 s); and a final extension at 72°C for 10 min.

Each PCR mixture contained 6.25 μL Multiplex Master Mix (Multiplex PCR Kit, QIAGEN, Germany), 2.5 μL ddH_2_O, 1.25 μL of each primer (10 μM solution), and 1.25 μL of extracted DNA, for a final volume of 12.5 μL. A positive control (*S. stercoralis* [Basso et al., 2019],) and a non-template control (NTC; RNase-free water) were included in each run.

Amplicons from each conventional PCR assay were analysed by capillary electrophoresis using the QIAxcel Connect Capillary Electrophoresis Instrument with a screening gel cartridge (QIAGEN, Germany). PCR products were then purified using the DNA Clean and Concentrator (Zymo Research, USA) according to the manufacturer's instructions with minor changes (all centrifugations for 1 min at 14,000 x g). DNA concentration and purity were assessed using a spectrophotometer (NanoDrop One, Thermo Scientific). Selected purified PCR products were submitted to Microsynth (Balgach, Switzerland; https://srvweb.microsynth.ch) for bidirectional Sanger sequencing, using the same primers employed in the corresponding PCR assays. Chromatograms were aligned and analysed using the Geneious Prime software (Version, 2026.0.1), and consensus sequences were compared with other sequences available in GenBank using BLASTn megablast and filtering the sequences with the highest identity.

### 2.6 Oxford Nanopore Technologies (ONT) deep amplicon sequencing

#### 2.6.1 Library preparation and MinION sequencing

Amplicon libraries were prepared using the Ligation Sequencing Amplicons - Native Barcoding Kit (SQK-NBD114.24; Oxford Nanopore Technologies [ONT]) following the manufacturer's protocol (version NBA_9168_v114_revR_30jan2025). For each individual felid sample, *18S* and *cox1* PCR products were pooled to achieve a starting DNA input of approximately 130 ng (∼65 ng per target) for each barcode. End-preparation, native barcoding, and adapter ligation were performed as directed by the manufacturer using commercial enzyme mixes (New England Biolabs). To strictly prevent magnetic bead carryover during the clean-up steps, AMPure XP bead elution volumes were conservatively reduced, retaining 10 μL of the cleared library. The final adapter-ligated library was quantified via Qubit fluorometry (ThermoFisher Scientific), and 65 ng of the library were loaded onto an R10.4.1 flow cell (FLO-MIN114) for sequencing on a MinION Mk1D device (ONT). Raw sequence data (.pod5 files) were acquired using the MinKNOW software. The sequencing run was monitored in real-time and terminated upon reaching approximately 300,000 reads per barcode.

#### 2.6.2 Bioinformatics and phylogenetic analysis

The offline basecalling of raw data was performed using the Super Accurate (SUP) model in Dorado v1.2.0 (ONT) with GPU acceleration, generating unaligned BAM files. Subsequent demultiplexing was executed using the Dorado demux function (configured for kit SQK-NBD114-24) to generate barcode-specific FASTQ files.

Custom bioinformatic pipelines were utilized to process the sequencing data for both the *18S* and *cox1* markers. Raw reads were initially filtered for primer presence (in any position and orientation), followed by primer and flanking sequence trimming using Cutadapt. A sequence length filter was applied using SeqKit (800–850 bp for *18S*; 880–940 bp for *cox1*). The retained reads were then dereplicated and subjected to *de novo* chimera removal using VSEARCH.

Operational Taxonomic Units (OTUs) were generated per barcode by clustering sequences with VSEARCH. Following the evaluation of OTUs represented by at least 50 reads, final similarity clustering thresholds were established at 98% and 99% for the *18S* and *cox1* pipelines, respectively. An overarching analysis was subsequently performed to cluster all filtered reads across all barcodes, quantifying the read distribution per OTU and sample. Basecalling, demultiplexing and pipelines were run using command-line tools in the Linux terminal (PC that used an ASUS TUF GeForce RTX 5080-16 GB-GPU and an Intel® Core™ i9-14900KS 24-core processor).

Representative OTU sequences were queried against the NCBI database using BLASTn megablast. Multiple sequence alignments of the generated OTUs and manually curated reference sequences were performed using Geneious Prime (Biomatters). Phylogenetic trees were constructed using the MrBayes plugin within Geneious Prime, designating *Parastrongyloides trichosuri* sequences (*18S*: AB923885; *cox1*: LC050209) as outgroup. To verify protein-coding integrity, the *cox1* OTU sequences were translated into all six possible reading frames using Geneious Prime.

## 3 Results

### 3.1 Microscopical examination and prevalence

In total, *Strongyloides*-like rhabditiform larvae were detected in samples from 30/490 felids (6.1%) by Baermann. Only one domestic cat sample (adult female) resulted positive for *Strongyloides* sp. larvae, resulting in a positivity rate of 0.6% (1/172). The sample was collected at necropsy in 2022 within the frame of a previous study (Furtado Jost et al., 2023). The intestinal content of the positive domestic cat was cultured for 1 week at room temperature and filariform L3, free-living males and females were recovered. Of all the samples analysed from wild felids, 29 were positive for *Strongyloides* spp. rhabditiform larvae, resulting in an overall prevalence of 9.6% (25/261) in lynx and 7.0% (4/57) in wildcats. All the positive samples were from free-ranging wild felids except one captive lynx. The prevalence in wild felids was significantly higher than in domestic cats (p ≤ 0.001).

**Fig. 1:**
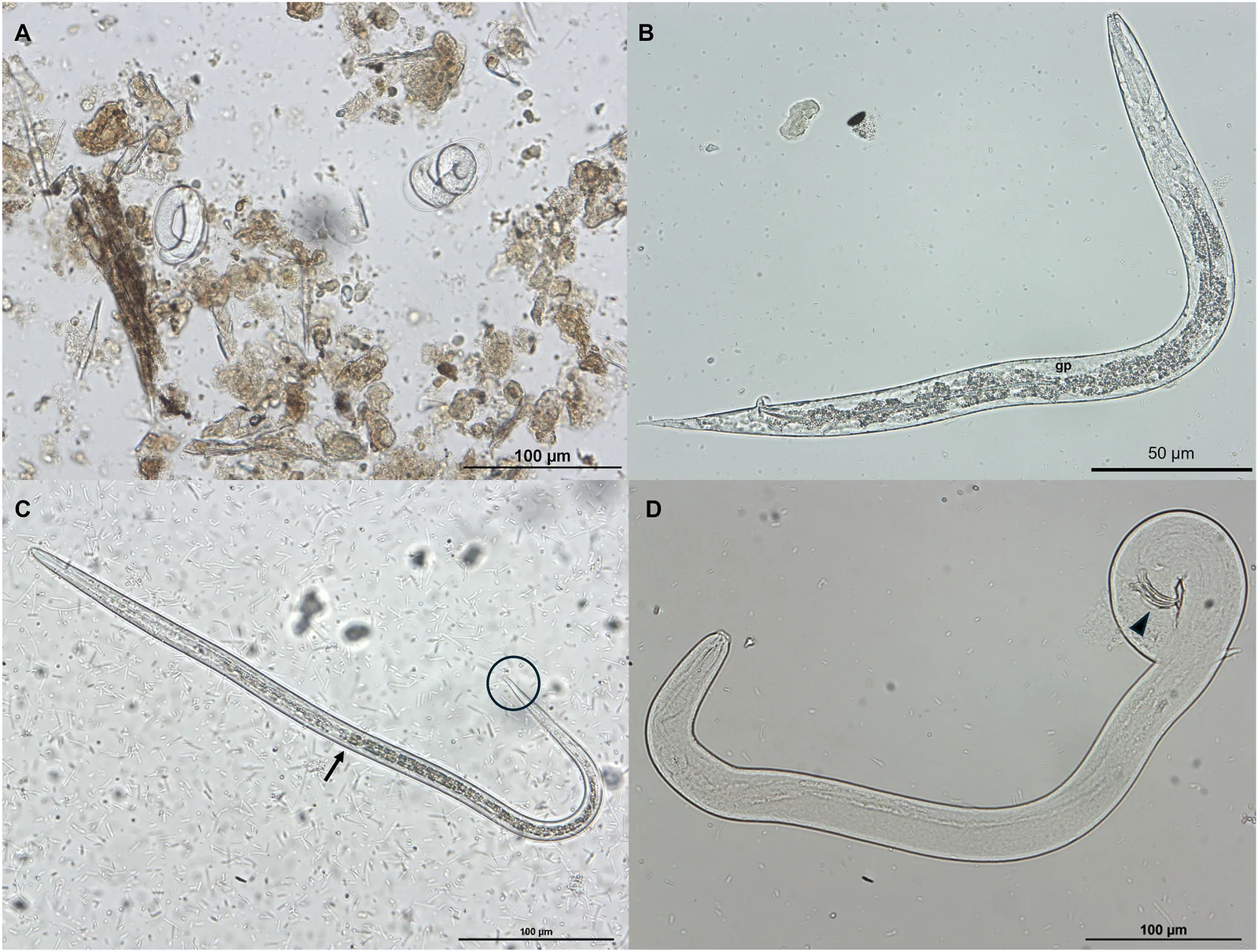
**A-D**: Photomicrographs of coproscopical examination by sedimentation flotation (**A**) and Baermann (**B-C**) methods. **A**: *Strongyloides* sp. eggs obtained from a Eurasian lynx (*Lynx lynx*) (21D348). **B**: *Strongyloides* sp. rhabditiform larva obtained from a European wildcat (*Felis silvestris*) (21D3333). Note the prominent genital primordium (gp). **C**: *Strongyloides* sp. filariform third-stage larvae (L3) from a lynx (21D348). Note the notched tail end (circle) and the long oesophagus (the arrow indicates the union with the intestine). **D**: *Strongyloides* sp. free-living male obtained from a lynx (21D348). Note the prominent spicule ventral membrane (arrowhead).

In eight positive lynx samples, *Strongyloides* sp. larvated eggs (measuring on average 55.7 × 33.4 μm) were additionally detected by the sedimentation-flotation technique (Fig. 1A).

In several positive samples, two groups of rhabditiform larvae were observed, measuring on average 268 × 17 μm (240.2-286.6 x 14.6-20.7 μm) and 325.4 × 17.9 μm (298.1-355.8 x 14.6-21.2 μm), respectively. All larvae contained a large genital primordium and a shallow buccal cavity (Fig. 1B). In addition, filariform L3, as well as rhabditiform males and females were detected by the Baermann technique in three free-range lynx samples. Filariform L3 were also detected in a positive captive lynx sample. The filariform L3 measured on average 570.7 × 16.8 μm (518.3-670.7 x 14.6-22 μm), with an oesophagus occupying 40-45% of the worm length and a typical notched tail end (Fig. 1C). Adult males measured on average 672.4 × 33.6 μm (562.2-792.7 x 22-42.9 μm), with a prominent spicule ventral membrane (Fig. 1D), and rhabditiform females 972.3 × 45.1 μm (868.9-1071.4 x 36.6-53.6 μm).

The positive lynx were 14 males and 11 females. *Strongyloides* spp. positivity by age group was 14/162 (8.6%), 5/35 (14.3%), 5/58 (8.6%) for juveniles, subadults and adults, respectively. For the positive captive lynx the age was not known. There were no significant differences in *Strongyloides* spp. positivity among the age groups. The positive wildcats were three females and one male; 1/7 juveniles and 3/43 adults. The low number of tested juvenile wildcats prevented further statistical comparisons.

**Fig. 2:**
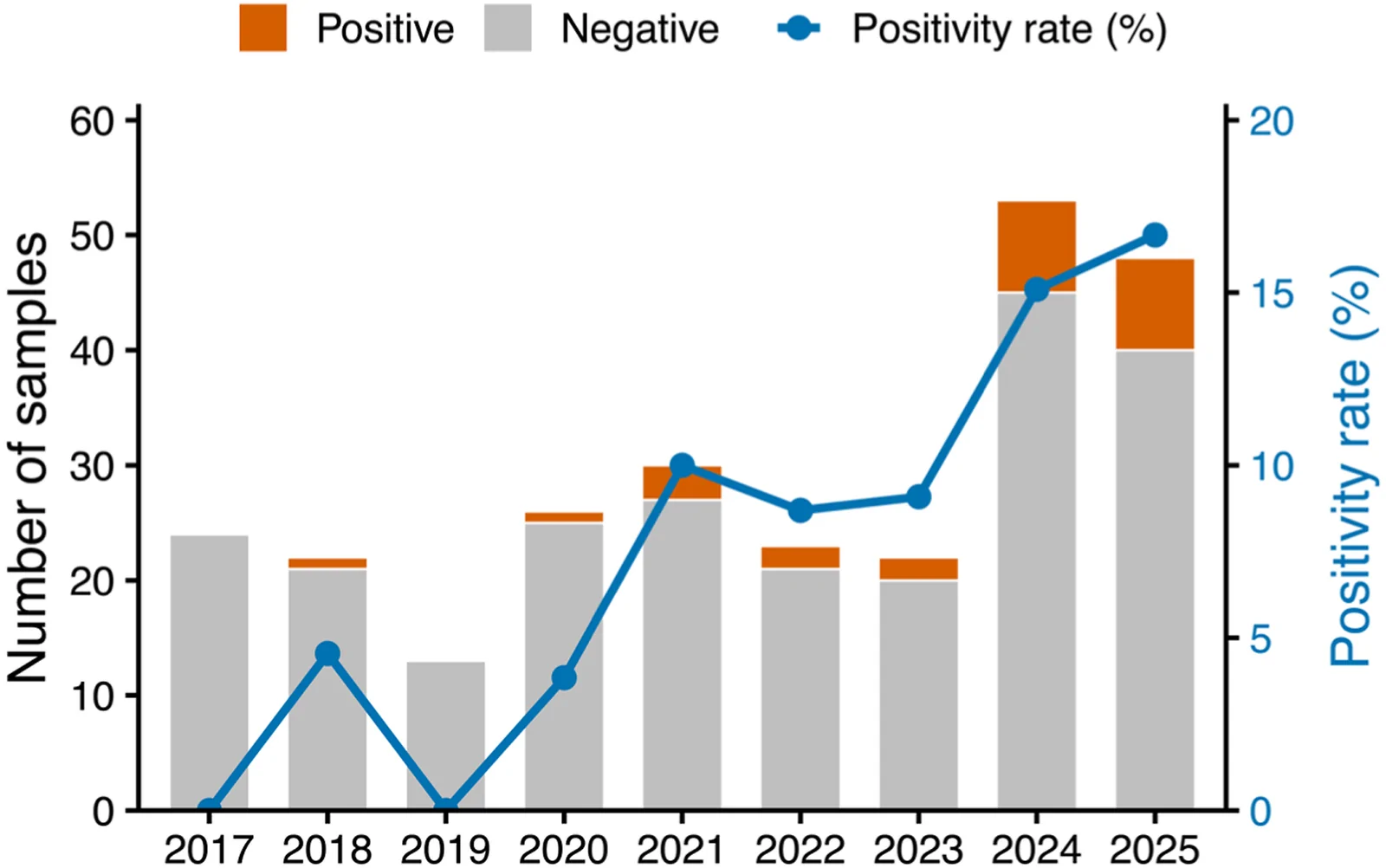
Temporal distribution of Eurasian lynx (*Lynx lynx*) tested for *Strongyloides* spp. through coproscopical examination. The blue line represents the average of *Strongyloides* spp. positivity over time.

**Fig. 3:**
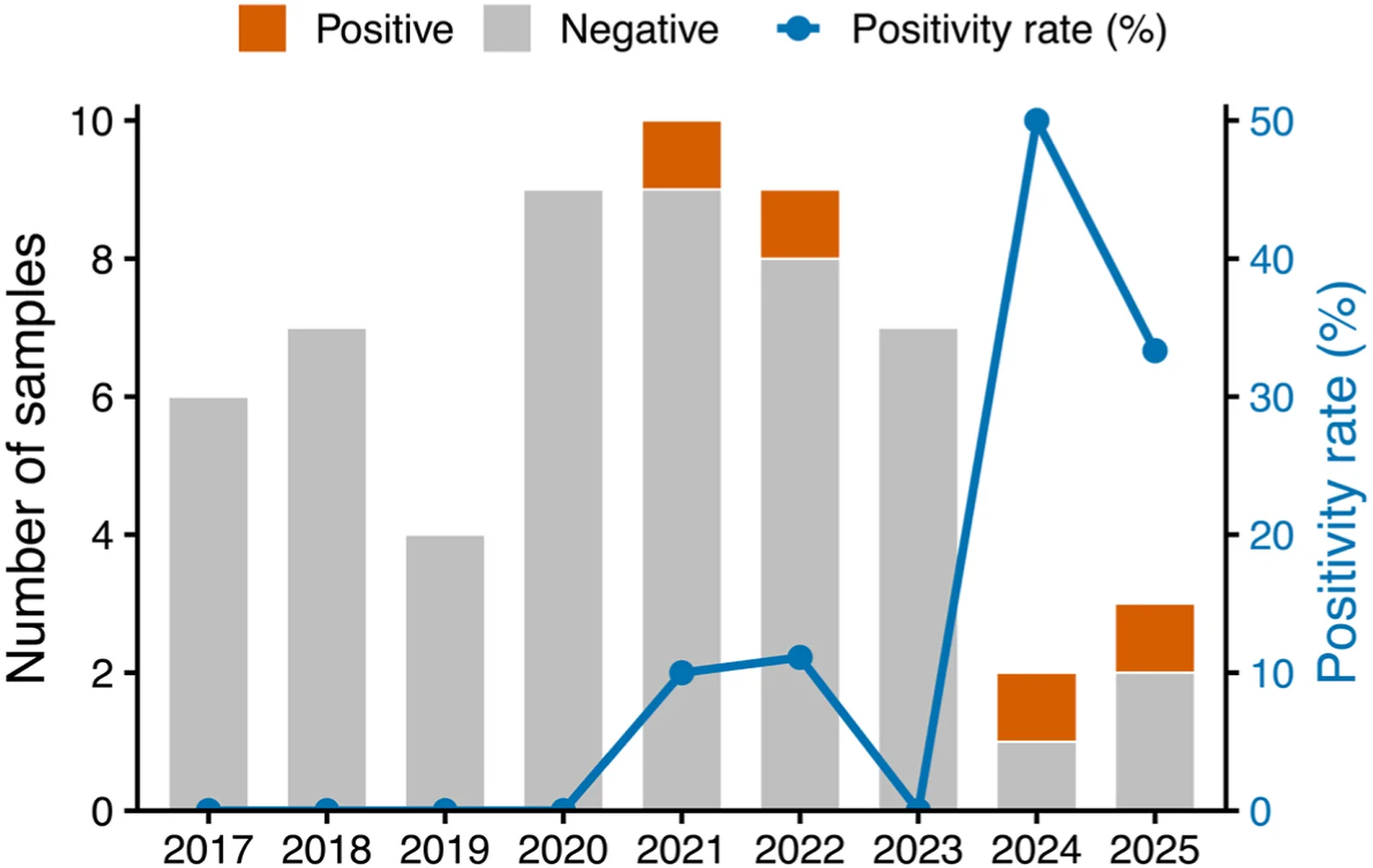
Temporal distribution of European wildcats (*Felis silvestris*) tested for *Strongyloides* spp. through coproscopical examination. The blue line represents the average of *Strongyloides* spp. positivity over time.

The first positive cases in wild felids were detected in 2018 (lynx) and 2021 (wildcats). Temporal distribution of the positive cases for wild felids is shown in Fig. 2, Fig. 3.

**Fig. 4:**
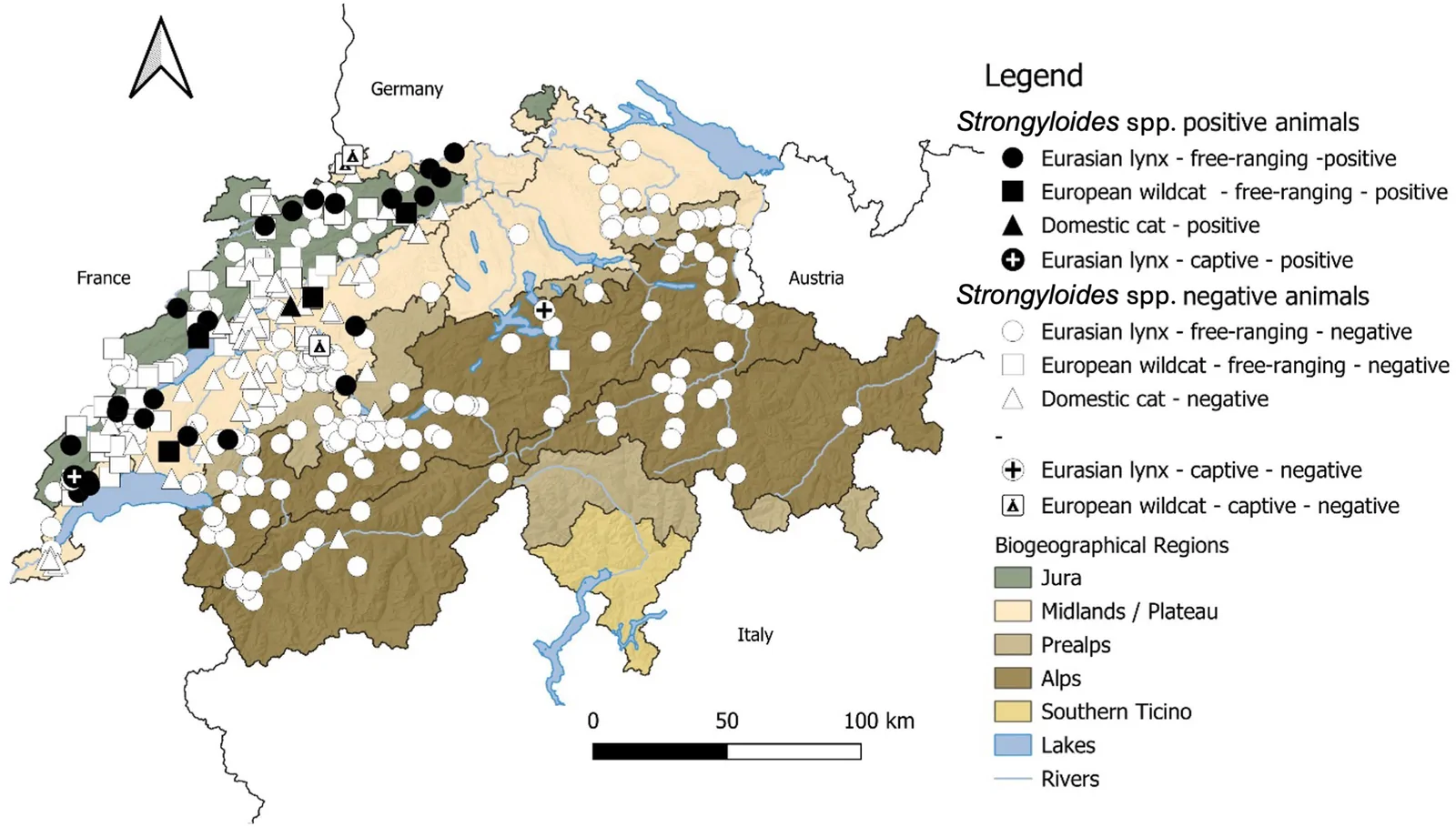
Origin of domestic cats (*Felis catus,* triangles), European wildcats (*Felis silvestris,* squares) and Eurasian lynx (*Lynx lynx,* circles) tested for *Strongyloides* spp. in Switzerland. For free-range wild felids, the geographical coordinates, where the carcasses were found or the animals were captured, are indicated. The different biogeographical regions of Switzerland are colour labelled.

Spatial distribution of domestic and wild felids tested and of positive cases for *Strongyloides* spp. in Switzerland are shown in Fig. 4.

Positive free-range lynx samples originated from the Jura (13/75), Midlands (10/62), and northern Prealps (1/26). None of the lynx samples from the Alps (n = 86) was positive for *Strongyloides* spp. The positive captive lynx was from a Zoo in the Jura region. The proportion of positive free-range lynx from the Jura-Midlands regions (23/137; 16.8%) was significantly higher than that from the Prealps-Alps regions (1/112; 0.9%) (p < 0.0001). Positive wildcats originated from the Jura (2/32) and from the Midlands (2/20), and the only sample from the biogeographical region Alps was negative. The only positive domestic cat originated from Midlands (Fig. 4).

All samples containing *Strongyloides* sp. were also positive concurrently for other parasites, including *Ancylostoma* sp. and other hookworms, *Capillaria* spp., *Cystoisospora felis*, *Dicrocoelium dendriticum*, *Eimeria* spp., *Felicola* spp., lungworms, *Taenia* spp. and *Toxocara cati*, representing either real infections or mere intestinal passage.

From 22/30 samples from felids containing *Strongyloides* spp. eggs/larvae, enough material was reserved for molecular studies (i.e., 17 lynx, all four wildcats and the domestic cat). For five animals, two to three sample replicates were processed, resulting in a total of 30 samples from 22 animals selected for subsequent molecular analyses.

### 3.2 Necropsy and histopathology

Positive free-range lynx showed a good to very good (n = 14), mildly to moderately reduced (n = 4), or severely reduced body condition (n = 6), respectively. Positive wildcats showed a good to very good (n = 3), or a severely reduced body condition (n = 1), respectively. The level of autolysis of the lynx organs was considered light (n = 13), moderate (n = 7) or severe (n = 4). The level of autolysis of the wildcat organs was considered light in all four animals. The features of the intestinal mucosa could only be partially identified. In one wildcat, sections of a structure compatible with *Strongyloides* sp. larvae could be detected in the mucosa. In none of the examined lung samples and of the small intestine sections of lynx, structures compatible with *Strongyloides* sp. could be detected.

### 3.3 Polymerase chain reactions (PCRs) and Sanger sequencing

*18S*: All Sanger sequencing attempts of the HVR IV region failed, producing either no sequence, chromatograms with superimposed peaks, or short sequences from other non-targeted organisms (fungi, other nematodes, etc.).The HVR I yielded positive results in 27/30 samples from 21/22 positive animals. Only three samples from lynx produced a clear consensus sequence (818 bp, primers trimmed). The consensus sequences were identical to one another, and showed a 100% identity with a *S. planiceps* sequence (LC536168 of 508 bp) reaching a 62% coverage or overlapping with our longer sequence), or 97% identity (100% coverage) with *S. robustus* (AB272232) and *S. callosciureus* sequences (AB272230). The remaining sequences were short, not readable and/or showed superimposed peaks in the chromatograms.

*cox1*: The samples processed with the primer combination COI int F- COI int R resulted in negative or weak positive results, and the sequencing attempts failed. Afterwards, three samples (21D348B, 21D820 and 21D927) were tested using the other two different primer combinations (Ko et al., 2020) to assess PCR product quality. Both PCR assays produced positive results; however, weaker products and more unspecific bands were observed when using the ENM283 reverse primer. Only two moderate quality consensus sequences were obtained. Both sequences (902 and 878 bp, trimmed by quality) were almost identical (only two SNPs where unresolved nucleotides were located) and showed a 99.46% identity with a *S. planiceps* sequence (PX148869 of 556 bp rendering in only 63% coverage with our sequences) and 87.2% identity (100% coverage) with a *Parastrongyloides trichosuri* sequence (NC_028620). Given the overall quality of the PCR products, it was decided to process all samples using primers ENM282*-*ENM310. In total, 23/30 samples were successfully amplified.

Due to the low quality and resolution of the Sanger reads, this approach was discontinued, and an ONT deep amplicon sequencing method was subsequently evaluated. As some samples represented duplicates from the same animal, the sample yielding the highest-quality PCR product from each animal was selected. Overall, 17 samples (13 lynx, three wildcats and one domestic cat) with both targets amplified (*18S* HVR I and *cox1* with primers ENM282*-*ENM310) were chosen for subsequent ONT sequencing.

### 3.4 ONT deep amplicon sequencing and phylogenetic analysis

Amplicon libraries generated approximately 5.3 million reads over 18 h of sequencing. All barcodes were detected, with an average of 335,000 reads per barcode. The bioinformatic pipelines were run multiple times for both loci using different similarity thresholds and minimum read cut-offs to polish the results. One of the lynx samples (24D4268) showed no reads after processing with the different pipeline setups.

**Fig. 5:**
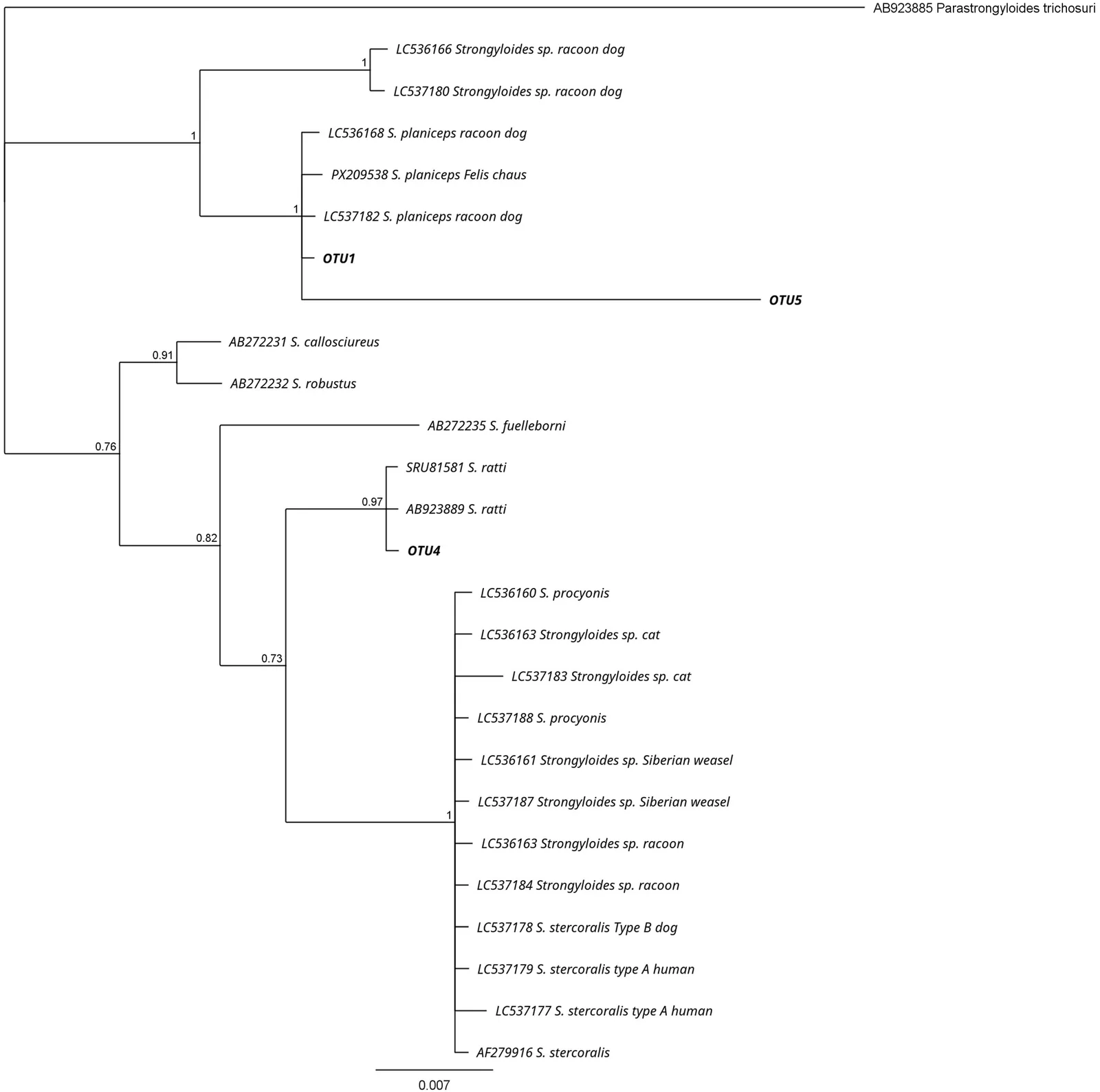
Phylogenetic tree based on a multiple sequence alignment of the 3 *Strongyloides* sp. OTUs (with more than 10,000 reads) for *18S* HVRI obtained in the present study (**in bold**) along with *Strongyloides* spp. *18S* reference sequences. The figure represents the posterior output of the MrBayes Plugin of Geneious Prime Software. A *18S* sequence of *Parastrongyloides trichosuri* sequence (AB923885) was used as the outgroup. Numbers at nodes are Bayesian posterior probabilities.

*18S*: The pipeline was initially run using a similarity threshold of 97%, retaining representative OTU sequences with at least 100 reads overall. This analysis resulted in more than 2.2 million reads clustered in 167 OTUs, of which 19 corresponded to *Strongyloides* spp., 122 to other nematodes, and 26 to fungi, bacteria, algae, or protozoa. Due to the high number of OTUs assigned to non-target species, subsequent OTU analyses were performed. For the last improved run, the pipeline was set with a higher similarity threshold (98%), and only OTUs represented by at least 10,000 reads overall were retained. This analysis yielded five OTUs clustering about 1.88 million reads (Table 1). Among these, only the first and most abundant OTU was detected in all 16 samples, accounting for a total of 1,623,885 reads. This OTU had a sequence length of 818 bp, and BLAST analysis revealed 100% identity with a *S. planiceps* sequence (LC536168 of 508 bp, 62% coverage). The remaining four OTUs were present at much lower abundances. Specifically, the second and third most abundant OTUs showed 99.88% identity with an *Uncinaria stenocephala* sequence (MN218457) and 100% identity with a *Troglostrongylus* sp. sequence (GU946677), respectively. The fourth OTU was detected in only one sample and showed 100% identity with a *S. ratti* sequence (U81581), while the fifth OTU consisted of 14,596 reads and showed 96.85% identity (62% coverage) with a *S. planiceps* sequence (LC536168) (Table 1). The OTU 1 and 5 contained the HVRI motif reported as *S. planiceps* from raccoon dogs from Japan (Ko et al., 2020). Subsequently, phylogenetic analysis performed by Geneious using the three OTUs with high identities with *Strongyloides* (OTU 1, 4 and 5) and several *18S* sequences from other *Strongyloides* spp., revealed that both OTU 1 and 5 positioned in a well-resolved branch together with other *S. planiceps* sequences (Fig. 5).

**Table 1:** Operational Taxonomic Units (OTUs) obtained through ONT deep amplicon sequencing of the *18S* HVR I PCR with more than 10,000 total reads across all samples. References: (L) = Eurasian lynx (*Lynx lynx*); (W) = European wildcat (*Felis silvestris*); (C) = Domestic cat (*Felis catus*). (−): < 50 reads.^1^ = the sequence reported in the GenBank is shorter (508bp), and for the OTU5 the homology was 492/508 bp.

	OTU 1	OTU 2	OTU 3	OTU 4	OTU 5	
BLAST Identity	100%	99.88%	100%	100%	96.85%	
BLAST Coverage	*62%*^1^	*96%*	*97%*	*100%*	*62%*^1^	
Length bp	818	845	846	817	818	
Reference sequence	LC536168	MN218457	GU946677	U81581	LC536168	Total Reads
Species Sample ID	*Strongyloides planiceps*	*Uncinaria stenocephala*	*Troglostrongylus* sp.	*Strongyloides ratti*	*Strongyloides planiceps*	
18D882 (L)	146,666	-	-	-	1009	147,675
20D3281 (L)	17,051	-	-	-	6761	23,812
21D348B (L)	140,990	10,765	-	-	-	151,755
21D820 (L)	228,822	-	-	-	1513	230,335
22D3572 (L)	20,680	-	-	-	146	20,826
23D4071 (L)	29,397	141,896	-	-	182	171,475
24D3816 (W)	67,178	-	-	-	544	67,722
24D4050 (L)	165,764	-	-	-	850	166,614
25D1296 (L)	126,639	-	-	-	215	126,854
25D1408 (L)	198,188	-	-	-	963	199,151
25D3386 (L)	77,891	-	330	-	396	78,617
25D3460 (L)	100,562	-	57,034	-	636	158,232
PVK25 (C)	72,346	3916	-	-	580	76,842
25D4088 (W)	113,893	7725	-	-	-	121,618
22D2917 (W)	43,915	-	-	15,509	204	59,628
24D1413 (L)	73,903	-	-	-	597	74,500
Total Reads	1,623,885	164,302	57,364	15,509	14,596	1,875,656

On the other hand, the OTU 4, positioned in a separate, well-supported branch together with *S. ratti* sequences. The OTUs 1, 4 and 5 belonging to *Strongyloides* spp. were submitted to GenBank (Accession numbers PZ744376-PZ744378).

*Cox1*: The initial pipeline was run using a similarity threshold of 96 and 97%, retaining only OTUs with at least 100 reads overall. These analyses generated more than 200 OTUs (about 1.5 million reads) and included sequences with similarities with *Strongyloides* spp. sequences, but also fungal and other non-target organisms. The final selected analysis was run using a pipeline set on 99% of similarity threshold, retaining only OTUs with at least 1000 overall reads. This analysis generated 72 OTUs clustering about 1 million reads. Of these, 69 OTUs showed varying levels of identity with a *S. planiceps* sequence (PX148869 from a jungle cat, *Felis chaus,* from Iran, unpublished) with low coverage (around 61% due to the shorter GenBank sequence), two OTUs were identified as fungi, while one OTU had a 97% identity and 100% coverage with a *S. ratti* sequence (NC028623). When only OTUs with more than 10,000 reads overall were considered, 16 OTUs were retained represented by sequences ranging from 910 to 914 bp in length (Table 2). None of these OTUs was detected in all samples. Fifteen out of 16 OTUs showed the highest BLAST identity with the same sequence of *S. planiceps* (PX148869), while one showed the highest identity (97%) with *S. ratti* (Table 2). Fourteen of these OTUs were coding, 13 for an identical protein sequence (one with a predicted single amino acid difference) and the one of *S. ratti* showed 32-to 40 amino acid differences. Two of the sequences were non-coding (OTU10 and 12). The remaining 56 OTUs (sequences ranging from 907 to 918 bp), clustering between 1000 and 9573 reads, contained the two sequences identified as fungal and 54 with identities ranging from 97.5 to 99.1% (60-61% coverage) with a *S. planiceps* sequence (PX148869). Of these, 32 OTU sequences contained stop codons or were non-coding.

**Table 2:** Operational Taxonomic Units (OTUs) obtained through ONT deep amplicon sequencing of the *cox1* fragment with more than 10,000 total reads across all samples. References: *: Reads values are expressed as ×10^3^; (L) Eurasian lynx (*Lynx lynx);* (W) = European wildcat (*Felis silvestris*); (C) = Domestic cat (*Felis catus*). (−): < 50 reads. Reference sequence: 1 = PX148869, reported as from *Strongyloides planiceps* from a jungle cat (*Felis chaus)* from Iran, 556bp. Since this sequence is shorter, the identity of the OTUs in the overlapping fragment range from 545 to 552 of 556 bp. 2 = NC_028623, *Strongyloides ratti* from Winstar rats.

OTU	1	2	3	4	5	6	7	8	9	10 nc	11	12 nc	13	14	15	16	
BLAST Identity%	98.5	99.2	99.1	98.9	98.9	98.7	99.3	98.5	98.5	98.5	98.2	98.9	98.5	97.0	98.9	98.0	
Coverage%	61	61	61	61	61	61	61	61	61	61	61	61	61	100	61	61	
Length (bp)	912	912	912	912	912	912	912	912	912	910	912	914	912	912	912	912	
Reference sequence Sample ID	1	1	1	1	1	1	1	1	1	1	1	1	1	2	1	1	Total *
18D882 (L)	5,4	0,3	3,4	18,4	1,6	14,3	-	1,0	3,2	1,5	2,8	1,5	3,6	-	-	-	57
20D3281 (L)	12,5	8,0	3,6	7,4	12,9	0,1	4,0	0,8	0,9	-	6,0	1,0	-	-	0,1	-	57,3
21D348B (L)	11,5	5,6	1,5	5,0	0,2	-	8,4	-	3,3	7,3	4,2	2,7	-	-	0,7	-	50,4
21D820 (L)	7,0	5,2	3,2	17,3	9,0	-	1,8	5,3	0,3	-	-	0,6	0,7	-	-	0,1	50,5
22D3572 (L)	26,4	2,3	0,4	0,7	0,7	0,4	3,3	0,3	0,1	0,4	0,2	0,2	-	-	-	-	35,4
23D4071 (L)	-	85,6	-	1,1	-	-	-	-	-	-	-	-	-	-	-	-	86,7
24D3816 (W)	5,1	0,2	1,4	15,1	1,1	1,0	0,1	-	3,2	1,6	1,2	1,2	-	-	-	-	31,2
24D4050 (L)	-	-	63,6	-	-	-	-	-	-	-	-	-	-	-	-	-	63,6
25D1296 (L)	65,0	-	1,1	-	18,4	-	-	-	-	-	-	-	-	-	-	-	84,5
25D1408 (L)	0,3	-	2,5	-	5,8	0,7	0,8	-	1,0	-	-	2,3	-	-	5,1	9,5	28,0
25D3386 (L)	5,8	1,7	3,7	1,6	4,6	-	1,2	0,3	4,1	4,8	1,4	2,5	2,0	-	2,5	-	36,2
25D3460 (L)	12,7	1,8	1,9	1,8	1,7	0,3	1,2	0,3	1,8	0,8	1,0	1,5	1,6	-	1,1	-	29,5
PVK25 (C)	12,3	-	1,3	-	4,7	6,8	-	12,8	1,1	-	-	2,2	6,1	-	-	0,2	47,5
25D4088 (W)	58,6	-	0,8	-	1,1	-	0,1	-	0,2	2,2	-	0,5	-	-	-	-	63,5
22D2917 (W)	-	12,2	-	7,8	0,1	-	0,5	-	-	-	-	-	-	14,2	-	-	34,8
24D1413 (L)	7,3	0,7	1,9	0,8	0,7	0,3	0,6	0,8	0,1	0,2	0,2	0,1	0,2	-	0,6	0,2	14,7
N Reads*	229,9	123,6	90,3	77,0	62,6	23,9	22,0	21,6	19,3	18,8	17,0	16,3	14,2	14,2	10,1	10,0	770,8

**Fig. 6:**
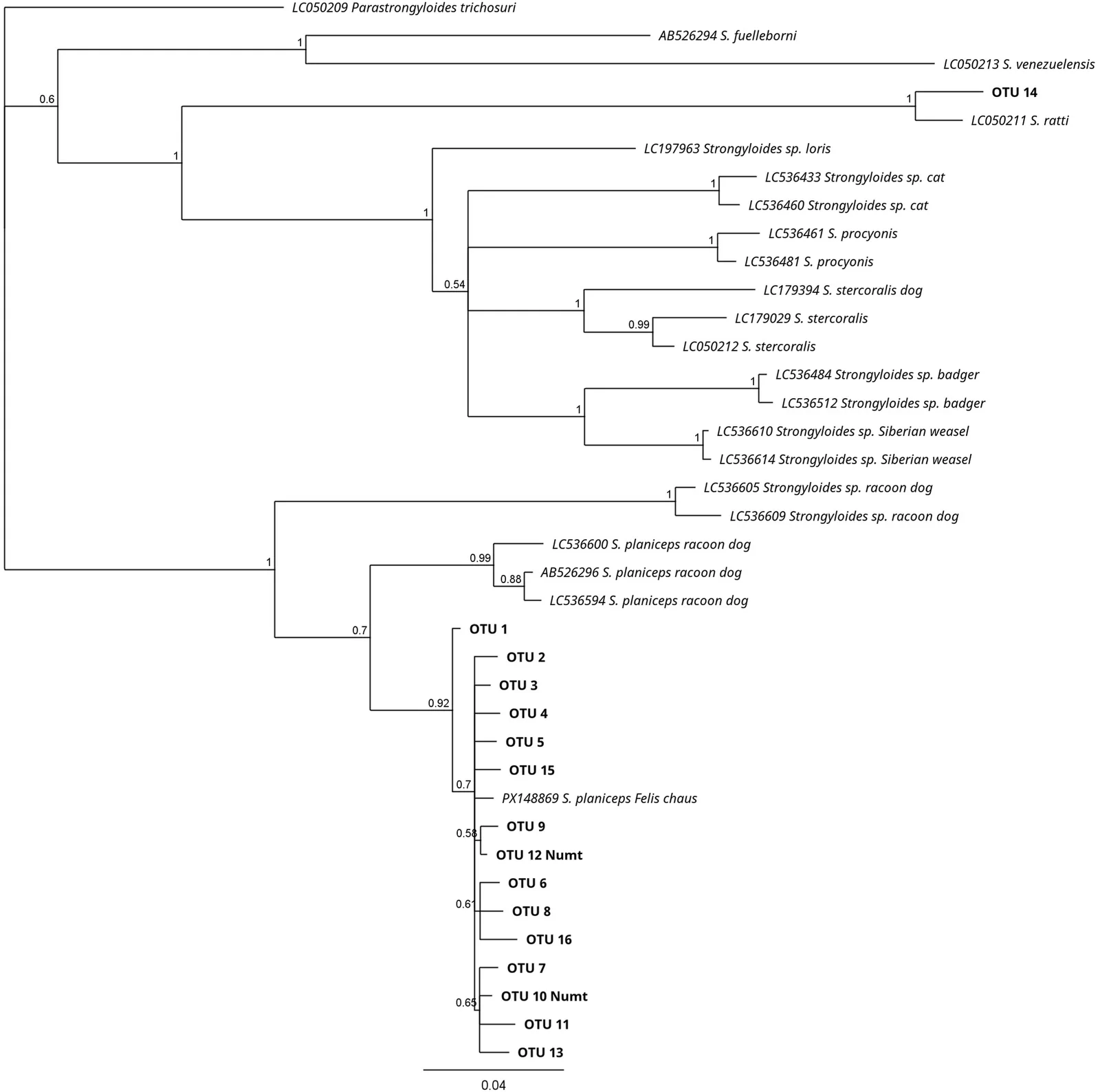
Phylogenetic tree based on a multiple sequence alignment of the 16 OTUs (with more than 10,000 reads) for the cytochrome *c* oxidase subunit I gene of mitochondrial DNA (*cox1*) obtained in the present study (**in bold**) along with *Strongyloides* spp. *cox1* reference sequences. The figure represents the posterior output of the MrBayes Plugin of Geneious Prime Software. A *cox1* sequence of *Parastrongyloides trichosuri* sequence (LC050209) was used as the outgroup. Node numbers are Bayesian posterior probabilities.

In the phylogenetic tree, 15 *cox1* OTUs were positioned altogether in a clade along with the sequence PX148869 reported as *S. planiceps* from a jungle cat (*Felis chaus*), and in a branch with *S. planiceps* sequences from raccoon dogs as a sister clade. The OTU 14, positioned in a separated and well supported branch together with a *S. ratti* sequence (Fig. 6). The coding *cox1* OTUs with more than 10,000 reads were submitted to GenBank (Accession numbers PZ744475-PZ744488).

## 4 Discussion

Epidemiological data on feline strongyloidosis are limited and disparate, with the prevalence and distribution of given *Strongyloides* spp. poorly understood. A recent meta-analysis for pooled *Strongyloides* prevalence in felines, identified 40 studies worldwide with sufficient information (Zhao and Bradbury, 2024). Most of these studies were performed in domestic cats and identified the parasites only to the genus level. Moreover, only two studies, both conducted in domestic cats, properly identified the species, with *S. felis* being detected*,* based either on morphology only (Speare and Tinsley, 1987) or on morphological and molecular features (Jitsamai, 2019). To date, no molecular epidemiological studies on *Strongyloides* spp. infections in wild felids have been performed (Zhao and Bradbury, 2024).

We hereby conducted the first combined morphological and molecular identification of *Strongyloides* spp. in both domestic cats and wild felids from Europe, namely in Eurasian lynx and European wildcats and assessed their prevalence in Switzerland. Noteworthy, we have performed a combination of coproscopical methods (sedimentation-flotation and Baermann), allowing for the detection of both larvated eggs and free larvae, thus reaching a more accurate estimation of the prevalence (Zhao and Bradbury, 2024). The *Strongyloides* spp. prevalence in wild felids in Switzerland, most of which were free-ranging, was 9.2% (29/318), being slightly higher in lynx 9.6% (25/261) than in wildcats 7.0% (4/57). The overall prevalence was lower than the estimated pooled global prevalence in wild felids (20%) (Zhao and Bradbury, 2024). The prevalence in domestic cats was significantly lower (0.6%), with only one positive cat. Domestic pet cats in Switzerland roam in a more controlled environment, with potentially reduced transmission opportunities, and have access to veterinary care and preventive and curative use of anthelmintic drugs, possibly explaining the lower positivity (Furtado Jost et al., 2023, Zhao and Bradbury, 2024). Moreover, the prevalence of suburban or rural domestic cats was not assessed in the present study, which could be expected as higher or similar to the one reported for stray cats (Zhao and Bradbury, 2024). Nevertheless, and considering the opportunistic type of sampling together with a potential loss of parasitic structures after sample refrigeration, the detected prevalence could be an underestimation.

All but one positive sample originated from the Jura and the Midlands, with a single positive (lynx) sample from the northern Prealps region, close to the Midlands (Fig. 4). While for domestic cats and wildcats this distribution corresponded with the origin of most samples, this was not the case for lynx samples. In the latter species, most samples from free-ranging animals with defined origin (86/249) originated from the Alps, which hosts about two thirds of the Swiss lynx population (https://www.kora.ch/en/species/lynx/distribution). However, none of these samples was positive for *Strongyloides* spp. The lynx prevalence in the Prealps and Alps (0.9%) was significantly lower than in the Jura and Midlands (16.8%). While the connectivity between the lynx populations in the Alps and Jura Mountains is still limited, the number of territorial lynx in the Midlands has been increasing in recent years, thus blurring the boundary that used to separate these two core areas of lynx presence in Switzerland (Foundation KORA, 2021). The higher prevalence in Jura and Midlands could be associated with more favourable climatic conditions (higher temperature and humidity), which may promote the free-living cycle of *S. planiceps,* increasing environmental contamination (Thamsborg et al., 2017; Basso et al., 2019; Viney and Morris, 2022). In the Alps, the harsher climate and the lower abundance of further potential environmental contamination sources, such as domestic and wildcats, could account for the absence of positive cases observed in Alpine lynx. Alternatively, a different susceptibility to *Strongyloides* spp. infection between Eurasian lynx belonging to the Alps and to the Jura populations cannot be completely ruled out.

We have observed an increase in the number of positive cases in both wild felid species over time, with the first cases in 2018 (lynx) and 2021 (wildcats), respectively. This may suggest the emergence and spread of strongyloidosis in Switzerland. It is possible that environmental conditions (temperature and humidity) are changing, allowing the parasites to multiply during their free-living cycle phase. Additionally, the slow spreading of other potential susceptible hosts, like the racoon dogs coming from Germany and Austria to Switzerland (https://apps.wildtier.ch/mammals/?animal=50), might also play a role in the environmental contamination.

We were able to statistically compare the positivity for *Strongyloides* spp. only among the different age groups of lynx (enough samples for statistical power), detecting no differences among juveniles, subadults and adults. This is in contrast with the general concept that *Strongyloides* spp. infections are more frequent in younger animals (Thamsborg et al., 2017). Opposite to this, a study performed in domestic cats in Australia showed increasing positivity to *S. felis* with the animal's age. This difference could be due to a different *Strongyloides* sp. infecting the lynx in Switzerland and/or to a different behaviour of the parasites in domestic versus wild felids. On the other hand, the similar positivity among age groups, and particularly between juveniles and adults, could also suggest a lack of protective immunity against *Strongyloides* sp. infection.

Interestingly, all detected positive animals in the present study contained rhabditiform larvae in their samples, and only 8 lynx samples additionally showed larvated eggs. Moreover, the *Strongyloides* sp. infecting felids in Switzerland was both morphologically and molecularly identified as *S. planiceps*. According to the original description and further studies and revisions, *S. planiceps* is considered to shed mainly larvated eggs, which require several hours to hatch and produce free larvae in culture (Rogers, 1939; Zhao et al., 2026). The measurements of eggs and rhabditiform larvae from the smaller larvae group we had obtained were consistent with those reported for *S. planiceps* (Rogers, 1939; Zhao et al., 2026). The second group of larvae, measuring ≥ 298 μm on average, was considered to represent L2 from the same *Strongyloides* species, since no other species (e.g., *S. stercoralis*) was found in molecular studies. We also detected filariform L3 in four lynx, and rhabditiform males and females in three of them. This might indicate that incubation and egg hatching occurred in all positive samples of the present study. All but one of the positive samples were from faecal content collected at necropsy from carcasses of free-ranging wildlife, mostly preserved refrigerated after detection, and the one sample from the lynx in captivity was collected from the environment and submitted for diagnosis after 48 h. Potentially, the time elapsed between death and necropsy (and sample submission) may have been long enough for eggs to hatch. In addition, all the Baermann tests were performed overnight, possibly facilitating the hatching and even moulting of larvae. This may also explain the detection of L3 and free-living adults in some animals, which may require hours or days to develop (Rogers, 1939; Zhao et al., 2026). The presence of L3 could also indicate an autoinfection as described for *S. stercoralis* and associated with a poor immunological control, but to confirm this, fresh faecal material would be needed (Thamsborg et al., 2017; Zhao et al., 2026). Most of the positive free-ranging wild felids exhibited good or mildly to moderately reduced body conditions, potentially suggesting a low implication of strongyloidosis in the general condition of these animals. Unfortunately, the small section of intestine used for the routine histopathological examination, along with the fast autolysis of the intestinal mucosa, prevented us from detecting any adult filariform female. Further studies performed with the aim to identify the parasite location could be conducted using intestinal mucosal scrapings, which were far beyond the aims of the present study.

The initially performed conventional PCRs and Sanger sequencing provided poor results for both molecular markers (*18S* and *cox1*). The *18S* rRNA HVR IV PCR proved to be highly unspecific, amplifying not only other nematodes, but also non-parasitic organisms, and did not result in a single *Strongyloides* sp. sequence. Thereafter, the *18S* HVR I was selected instead, using the primers described for *S. stercoralis* typing (Jaleta et al., 2017), consistently yielding higher PCR positivity, although banding was still present. Therefore, conventional PCRs and Sanger sequencing were not helpful in identifying feline *Strongyloides* species using mainly Baermann sediment containing larvae as starting material for the DNA extraction.

PCR-sequencing of total DNA extracted from complex biological samples such as faecal material, may require deep amplicon sequencing for proper results (Zhao et al., 2026). There are limited data in the literature regarding the use of ONT deep amplicon sequencing in Parasitology (Šlapeta et al., 2026). Particularly for *Strongyloides* spp., the most used method has been the Illumina sequencing (Zhao et al., 2026). One of the weaknesses of this sequencing is the relatively short fragment coverage, being challenging and difficult to assemble if amplicons are longer than 450bp or display several repeats (Barratt et al., 2019; Šlapeta et al., 2026). Newer technologies such as ONT can sequence longer fragments, offering greater taxonomic resolution and flexibility in primer site selection, overcoming the Illumina weakness (Huggins et al., 2024). Moreover, the newest Flow Cell (FLO-MIN114, R10 Version) and MinION device (Mk1D) used in the present study, reduce the previous higher error rate of ONT compared to short-read Illumina sequencing (Šlapeta et al., 2026), achieving >99% raw read accuracy (https://nanoporetech.com/document/chemistry-technical-document).

Due to the limited literature available, the sequencing analysis pipelines were developed and progressively refined by optimising sequence similarity thresholds and minimum read counts, with particular attention to the number of OTUs generated after each run (Šlapeta et al., 2026). From 5.3 million total reads, 3.7 million (69.8%) were retained for the *18S* HVR I (2.2 million) and *cox1* (1.5 million) datasets after primer and length filtering, chimera removal, and exclusion of OTUs with fewer than 100 reads. This success rate exceeded that reported for ONT deep amplicon sequencing of filarial nematodes using SUP basecalling (Huggins et al., 2024), likely reflecting improvements in sequencing accuracy with newer ONT chemistry and hardware. Unlike previous studies that relied on consensus sequence generation to compensate for higher error rates (Huggins et al., 2024), the high (>99%) accuracy achieved here allowed OTU-based analysis, preserving true sequence variability. The ONT approach generated high-quality sequences for 16 of the 17 samples with detectable amplicons for both *18S* HVR I and *cox1*. However, initial analyses using a ≥ 1000-read threshold produced numerous OTUs, including *Strongyloides* spp., other nematodes, and non-target bacteria and fungi. This likely reflects the relatively low specificity of the primers, explaining the failure to obtain clean Sanger chromatograms due to mixed amplicons. Given that all animals harboured multiple parasites, amplification of non-target taxa was expected, particularly with primers targeting conserved regions. The *cox1* primers (ENM282/ENM310; Ko et al., 2020) were more specific than the *18S* HVR I primers (SSU18A/SSU26R; Jaleta et al., 2017). Indeed, most low-abundant *18S* HVR I OTUs (<10,000 reads) corresponded to non-target organisms, while two of the five most abundant OTUs represented *Uncinaria/Ancylostoma* spp. and *Troglostrongylus* sp., consistent with coproscopical results. Although the approach successfully identified *Strongyloides* spp., sequencing non-target amplicons reduced efficiency (Huggins et al., 2024). Greater primer specificity or enrichment of target material before DNA extraction would likely improve sequencing efficiency and accuracy (Zhao et al., 2026; Šlapeta et al., 2026).

Considering only OTUs with >10,000 reads, 16 samples (12 from lynx, three from wildcats and one from a cat) were successfully characterized at both targets, yielding 5 *18S* HVR I and 16 *cox1* OTUs, respectively. Of these, 2/5 (*18S* HVR I) and 15/16 (*cox1)* had the highest identity to *S. planiceps* sequences. The low sequence coverage observed (60-62%) is likely explained by the longer amplicons generated in this study compared with the shorter *S. planiceps* reference sequences currently available in GenBank (Hasegawa et al., 2009; Ko et al., 2020).

The combined morphology, repeated detection across animals of the same OTUs, and phylogenetic placement support a provisional assignment to *S. planiceps*, while the limitations of partial loci and database annotations prevent definitive species confirmation.

For the *18S* HVR I marker, OTU 1, the most abundant, was detected in all 16 samples, whereas OTU 5 was detected in 14/16 samples. Both clustered with *S. planiceps* in the phylogenetic tree and contained the HVR I motif described for this species (Ko et al., 2020), suggesting they may represent over- and underrepresented copies of the *18S* gene within the same organism. This source of intragenomic variation appears to have been overlooked in previous genotyping studies (Zhao et al., 2026).

As expected for *Strongyloides* spp. (Barratt et al., 2019), substantially greater variability was observed in the *cox1* sequences. Although 15 OTUs showed varying levels of similarity to a GenBank *S. planiceps* sequence (PX148869) from a jungle cat (unpublished), they all clustered within a single sister clade to *S. planiceps* sequences from raccoon dogs in Japan, suggesting they represent *cox1* haplotypes of *S. planiceps*. The separation of these clades may reflect host- or geographically associated lineages, with one comprising raccoon dog isolates from Japan and the other including sequences from felids in Switzerland and possibly jungle cats from Iran. This observation warrants further investigation to clarify the identity and taxonomic status of *S. planiceps* in raccoon dogs. Despite the genetic variability, the predicted protein sequence of coding OTUs was quite conserved, being 12 identical and one displaying only one difference. This confirms both, the low error rate of the ONT sequencing and the plurality of haplotypes not being associated with changes in the coding protein. Moreover, 15 samples had sequences from two to 15 of the OTUs (Table 2), indicating a potential mix of genotypes or mitochondrial heteroplasmy, which could have produced the difficulties observed in the Sanger sequencing (Zhao et al., 2026). The lynx sample with only one *cox1* OTU (24D4050) corresponded to the animal from a zoo, from which the larvae (L3) were collected and washed in PBS before DNA extraction (and was one of the samples with a consensus sequence in the Sanger sequencing of the *18S* HVR I, data not shown). The washing step may have improved or “purified” the material prior to DNA extraction allowing a clear Sanger sequencing and resulting later in only one *cox1* OTU (Zhao et al., 2026). Nevertheless, the *18S* HVR I ONT sequencing of this sample also showed two OTUs, reinforcing the presence of two *18S* copies or variants in *S. planiceps* previously mentioned.

The intraspecific genetic diversity of *S. planiceps* has not been previously researched (Zhao et al., 2026). The present study evidenced a relatively high variability of the *cox1* gene fragment and a more conserved (but probably holding of at least two variants) of the *18S* fragment containing the HVR I. The genotyping using mitochondrial genes could face some risks to overestimate variability due to co-amplification of nuclear mitochondrial DNA fragments or pseudogenes (NUMTs) (Huggins et al., 2024). However, most of the OTUs with more than 10,000 reads presented here were coding for a protein fragment, and therefore, the variability was confirmed. The NUMTs are fragments of mitochondrial DNA that have been transferred and inserted into the nuclear genome and accumulated a considerable number of mutations, which can be recognized by the presence of stop codons (Huggins et al., 2024). In our broader analysis, two of the most represented and 32/56 of the less represented *cox1* OTUs (between 1000 and 9573 total reads) were non-coding sequences, and therefore, were considered NUMTs. In any case, the presence of these NUMT sequences in the PCR amplicons may also explain the difficulty in obtaining clean Sanger read sequences.

In one wildcat sample (22D2917), in addition to *S. planiceps*, one OTU per target showed 100% (*18S*) and 97% (cox1) identity to *S. ratti*. The lower *cox1* identity is consistent with the greater intraspecific variability reported for this marker (Barratt et al., 2019). This was interpreted as the passage of *S. ratti* larvae from a rodent prey, highlighting the difficulty of interpreting coproscopical findings when only rhabditiform larvae are detected. Consequently, some historical *Strongyloides* spp. diagnoses based solely on microscopy may have represented prey-derived larvae, potentially leading to an overestimation of prevalence (Barratt et al., 2019; Zhao and Bradbury, 2024).

In conclusion, this study demonstrates a moderate coproscopical prevalence of *Strongyloides* in Swiss wild felids, being slightly higher in lynx (9.6%) than in wildcats (7%), while it was significantly lower (0.6%) in domestic cats. The spatial-temporal distribution showed that almost all positive felids originate from the Jura and Midlands and that case numbers seem to be increasing over time. The ONT deep amplicon sequencing and the developed analyses in this study are pioneer in Parasitology. They enable identification of strengths and limitations of widely used genetic targets and PCR assays, while also confirming *S. planiceps* in all analysed felids. The combined use of coproscopical methods and ONT deep amplicon sequencing allowed the identification of *S. planiceps* as the most prevalent species in Swiss felid populations. The impact of strongyloidosis in felid health, particularly in endangered species, requires further research.

## CRediT authorship contribution statement

**Gastón Moré:** Writing – review & editing, Writing – original draft, Visualization, Software, Methodology, Investigation, Formal analysis, Data curation, Conceptualization. **Giulia Colangeli:** Writing – review & editing, Writing – original draft, Visualization, Software, Methodology, Investigation, Formal analysis, Data curation. **Simone R.R. Pisano:** Writing – review & editing, Visualization, Formal analysis. **Fatou Geissler:** Writing – review & editing, Visualization, Investigation. **Iris Marti:** Writing – review & editing, Resources. **Sinah Lückner:** Writing – review & editing, Software, Formal analysis. **Chahrazed Belhout:** Writing – review & editing, Software, Resources, Investigation. **Diana S. Gliga:** Writing – review & editing, Visualization, Formal analysis. **Caroline F. Frey:** Writing – review & editing, Validation, Resources, Funding acquisition. **Walter Basso:** Writing – review & editing, Supervision, Methodology, Funding acquisition, Conceptualization.

## Ethical statement

No animals were killed specifically for the purposes of this study.

## Funding

This work was supported by internal funding from the Institute of Parasitology, University of Bern and partially by the granted project MA25_17 “*Strongyloides stercoralis* – prevalence and co-infection in pet dogs and people in Switzerland” from Multidisciplinary Center for Infectious Diseases (MCID) of the University of Bern, Switzerland. Giulia Colangeli received a mobility fellowship from CIBERINFEC – CIBER *de Enfermedades Infecciosas* (CB21/13/00056), *Instituto de Salud Carlos* III (ISCIII), Ministry of Science and Innovation, and the European Union, under the program “*Ayudas a la Formación y Movilidad*”.

## Conflicts of interest

We Gastón Moré, Giulia Colangeli, Simone R. R. Pisano, Fatou Geissler, Iris Marti, Sinah Lückner, Chahrazed Belhout, Diana S. Gliga, Caroline F. Frey, Walter Basso, authors from the manuscript intitled “**Prevalence and molecular characterization of *Strongyloides* spp. in domestic and wild felids from Switzerland**” report no conflict of interest.
